# A susceptibility gene signature for ERBB2-driven mammary tumour development and metastasis in collaborative cross mice

**DOI:** 10.1016/j.ebiom.2024.105260

**Published:** 2024-07-26

**Authors:** Hui Yang, Xinzhi Wang, Adrián Blanco-Gómez, Li He, Natalia García-Sancha, Roberto Corchado-Cobos, Manuel Jesús Pérez-Baena, Alejandro Jiménez-Navas, Pin Wang, Jamie L. Inman, Antoine M. Snijders, David W. Threadgill, Allan Balmain, Hang Chang, Jesus Perez-Losada, Jian-Hua Mao

**Affiliations:** a Biological Systems and Engineering Division, Lawrence Berkeley National Laboratory, Berkeley, CA, USA; b Instituto de Biología Molecular y Celular del Cáncer (IBMCC-CIC), Universidad de Salamanca/CSIC, Salamanca, 37007, Spain; c Instituto de Investigación Biosanitaria de Salamanca (IBSAL), Salamanca, 37007, Spain; d Department of Hematology, Zhongnan Hospital of Wuhan University, Wuhan, Hubei, 430079, China; e Department of Gastroenterology, Nanjing Drum Tower Hospital, Affiliated Hospital of Nanjing University Medical School, Nanjing, Jiangsu, 210008, China; f Berkeley Biomedical Data Science Center, Lawrence Berkeley National Laboratory, Berkeley, CA, 94720, USA; g Department of Nutrition, Texas A&M University, College Station, TX, 77843, USA; h Department of Molecular and Cellular Medicine and Department of Biochemistry & Biophysics, Texas A&M University, College Station, TX, 77843, USA; i Helen Diller Family Comprehensive Cancer Center, University of California San Francisco, San Francisco, CA, 94158, USA

**Keywords:** Breast cancer, Collaborative cross mice, Tumour susceptibility, Gene signature, Prognosis, Treatment response prediction

## Abstract

**Background:**

Deeper insights into ERBB2-driven cancers are essential to develop new treatment approaches for ERBB2+ breast cancers (BCs). We employed the Collaborative Cross (CC) mouse model to unearth genetic factors underpinning Erbb2-driven mammary tumour development and metastasis.

**Methods:**

732 F1 hybrid female mice between FVB/N MMTV-*Erbb2* and 30 CC strains were monitored for mammary tumour phenotypes. GWAS pinpointed SNPs that influence various tumour phenotypes. Multivariate analyses and models were used to construct the polygenic score and to develop a mouse tumour susceptibility gene signature (mTSGS), where the corresponding human ortholog was identified and designated as hTSGS. The importance and clinical value of hTSGS in human BC was evaluated using public datasets, encompassing TCGA, METABRIC, GSE96058, and I-SPY2 cohorts. The predictive power of mTSGS for response to chemotherapy was validated *in vivo* using genetically diverse MMTV-*Erbb2* mice.

**Findings:**

Distinct variances in tumour onset, multiplicity, and metastatic patterns were observed in F1-hybrid female mice between FVB/N MMTV-*Erbb2* and 30 CC strains. Besides lung metastasis, liver and kidney metastases emerged in specific CC strains. GWAS identified specific SNPs significantly associated with tumour onset, multiplicity, lung metastasis, and liver metastasis. Multivariate analyses flagged SNPs in 20 genes (*Stx6, Ramp1, Traf3ip1, Nckap5, Pfkfb2, Trmt1l, Rprd1b, Rer1, Sepsecs, Rhobtb1, Tsen15, Abcc3, Arid5b, Tnr, Dock2, Tti1, Fam81a, Oxr1, Plxna2,* and *Tbc1d31*) independently tied to various tumour characteristics, designated as a mTSGS. hTSGS scores (hTSGSS) based on their transcriptional level showed prognostic values, superseding clinical factors and PAM50 subtype across multiple human BC cohorts, and predicted pathological complete response independent of and superior to MammaPrint score in I-SPY2 study. The power of mTSGS score for predicting chemotherapy response was further validated in an *in vivo* mouse MMTV-Erbb2 model, showing that, like findings in human patients, mouse tumours with low mTSGS scores were most likely to respond to treatment.

**Interpretation:**

Our investigation has unveiled many new genes predisposing individuals to ERBB2-driven cancer. Translational findings indicate that hTSGS holds promise as a biomarker for refining treatment strategies for patients with BC.

**Funding:**

The U.S. 10.13039/100000005Department of Defense (DoD) Breast Cancer Research Program (BCRP) (BC190820), United States; MCIN/AEI/10.13039/501100011039 (PID2020-118527RB-I00, PDC2021-121735-I00), the “10.13039/501100000780European Union Next Generation EU/PRTR,” the Regional Government of Castile and León (CSI144P20), European Union.

**Research in context**

**Evidence before this study**

While it is well known that genetic variations control susceptibility to ERBB2-driven breast cancer (BC), many genetic factors remain to be unearthed.

**Added value of this study**

Using a large cohort of mice with genetic diversity, we identified over a thousand genetic variations controlling ERBB2-driven tumour phenotypes, including organ-specific metastasis, which had not been previously reported. Moreover, we found evidence that human orthologs of mouse susceptible gene signature for ERBB2-driven cancer can be used to not only stratify human patients with BC into distinct prognostic groups independent of clinical factors and PAM50 subtypes across multiple cohorts, but also predict treatment responses independent of the MammaPrint score in the I-SPY2 cohort.

**Implications of all the available evidence**

Progress towards precision medicine in patients with cancer requires considering individual genetic variations. The susceptible gene signature discovered from our mouse study may serve as a biomarker for tailoring treatment to patients with BC.

## Introduction

Twenty to thirty percent of primary breast cancers (BCs) amplify/overexpress the epidermal growth factor receptor 2 (ERBB2, HER2, or NEU).^1^^,^^2^ These ERBB2+ tumours have more aggressive disease and poorer clinical outcome, and are more refractory to radiotherapy, chemotherapy, and hormone therapy.^3^, ^4^, ^5^, ^6^ Although a humanized anti-ERBB2 monoclonal antibody (Herceptin) and the small molecule inhibitor of ERBB2 (Lapatinib) are effective for treating patients with ERBB2+ BC, most ERBB2+ BCs do not respond to either Herceptin or Lapatinib (intrinsic resistance), and the majority of responders become resistant within 12 months of initial therapy (acquired or secondary resistance).^7^, ^8^, ^9^, ^10^, ^11^ Therefore, new biological insights into HER2-driven cancers are still needed.

Our previous F1 backcross (F1Bx) study between the resistant C57BL/6J strain and FVB/N has shown a strong genetic effect on ERBB2-initiated tumour development and metastasis.^12^ Moreover, our omics analysis of tumours revealed similarities between ERBB2 tumours in humans and those from F1Bx mice at clinical, genomic, expression, and signaling levels.^12^ However, an obvious limitation of this F1Bx study is that we only found genetic variants between C57BL/6J and FVB/N and likely missed variants relevant to more diverse human populations. The Collaborative Cross (CC) mouse resource, established from 8-parental recombinant inbred mouse strains, contains uniformly distributed natural variants and a level of genetic diversity on a par with the human population.^13^, ^14^, ^15^ Moreover, large CC strain-dependent variations in many phenotypes, such as spontaneous tumour development, have been reported.^16^, ^17^, ^18^, ^19^, ^20^, ^21^, ^22^, ^23^, ^24^, ^25^, ^26^, ^27^, ^28^, ^29^

In this study, we identified host genetic variants that predispose *Erbb2*-driven tumour development and metastasis using the CC mouse resource. Additionally, we systematically evaluated the clinical value for prognosis and therapeutic responses of a mouse mammary tumour susceptibility gene signature in human BC using publicly available cohorts, including clinical trial cohorts. Our findings substantially increase biological insights into ERBB2-driven cancers, which may provide new strategies and define new targets for improving outcomes of ERBB2-targeted therapies.

## Methods

### CC mice experiments

All CC mice were purchased from the Systems Genetics Core Facility at the University of North Carolina, and FVB-Tg (MMTV-Erbb2)NK1Mul/J (FVB/N MMTV-*Erbb2*) mice were purchased from the Jackson Laboratory. F1 hybrid mice were generated by crossing FVB/N MMTV-*Erbb2* female mice with CC male mice from 30 CC strains. The number of female mice used in this study was summarized in Supplementary Table S1. 20 FVB/N MMTV*-ErbB2* female mice served as control. All mice were monitored for mammary tumour development by palpating with a maximum follow-up of 2 years. This study was approved by the Animal Welfare and Research Committee at Lawrence Berkeley National Laboratory (271004).

### Chemotherapy experiment in a genetically diverse MMTV-Erbb2 model

Genetically diverse F1 backcross (F1Bx) mice between C57BL/6J and FVB/N MMTV-Erbb2 transgenic mice were generated as described in our previous study.^12^ 50 Erbb2-positive F1Bx mice were housed at IBMCC-FICUS's Animal Research Facility and observed twice a week for tumour manifestation. Before starting treatment (when tumour volume reached 500 mm^3^), two biopsies were collected under aseptic conditions, in a flow chamber, and with isoflurane anesthesia. One biopsy was frozen for transcriptional analysis, and the other was fixed for histological analysis. Two weeks after collection of the tumour biopsy, mice underwent chemotherapy consisting of 5 intraperitoneal injections of 25 mg/kg docetaxel with a recovery time of 8 days between injections.

We evaluated the local tumour by assessing changes in the tumour growth. Tumour volume was estimated each week using the formula: Tumour volume = length x width^2^ x 0.5. We quantified tumour volume changes and growth rate. We calculated the tumour growth rate by estimating the linear regression slope of the logarithm of tumour volume in mm^3^ onto time in days. We defined (a) complete response (nonpalpable mammary tumour), (b) partial response (tumour volume significantly reduced at the end of treatment in comparison to the volume at the beginning of treatment), (c) tumour stabilization (no change in tumour volume during treatment in comparison to the volume at the beginning of treatment); and (d) early resistance (increase in tumour volume during treatment in comparison to the volume at the beginning of treatment) to therapy. All mice were housed at the Animal Research Facility of the University of Salamanca for mouse chemotherapy. All the procedures were approved by the Institutional Animal Care and Bioethical Committee of the University of Salamanca (PLE2009-0119).

### Genome-wide association study (GWAS)

GWAS analysis has been described previously.^19^^,^^26^, ^27^, ^28^ At each SNP, Cox regression was used to assess the significance of associations between tumour onset and allele types; the Mann–Whitney test was used to determine the significance of associations between tumour multiplicity and allele types; while the Chi-square test was used to assess the significance of associations between tumour metastasis (overall, lung, liver, and kidney metastasis) and allele types. Putative candidate genes were defined as those genes containing a significant SNP within the boundaries of the gene sequence (http://www.informatics.jax.org/). KEGG pathway enrichment analysis was performed on candidate genes using WebGestalt (https://www.webgestalt.org/).^30^

### RNA extraction from tumours

The Qiagen miRNeasy Mini Kit-50 was used for RNA extraction, preserving miRNA populations for further studies. The protocol followed was as previously described.^12^ Global RNA expression was assessed using Affymetrix chips at the University of Salamanca's Cancer Research Center's Genomics Unit or using RNA-seq analysis at the UCLA Technology Center for Genomics & Bioinformatics.

### RNA sequencing analysis

The Illumina sequencing platform (HiSeqTM 2500) was used to generate 150bp paired-end reads. RNA-sequencing reads were mapped to the mouse genome (GRCm38/mm 10 reference) using the align function in the Rsubread package (version 2.0.1) with default parameters. For each replicate, per-gene counts of uniquely mapped reads were computed using the featureCounts function in the Rsubread package (version 2.0.1). RNA-Seq data are available in the National Center for Biotechnology Information (NCBI) BioProject Repository (https://www.ncbi.nlm.nih.gov/bioproject) under the BioProject “PRJNA1122577”.

Mice were stratified using consensus clustering (ConsensusClusterPlus package in R, version 1.50.0) with hierarchical clustering, Pearson's correlation, and 1000 bootstrapping iterations at 90% sampling rate, and the optimal number of subtypes was determined by the consistency of cluster assignment (i.e., the consensus matrix).

### Gene expression profiling and analysis

RNA integrity was evaluated using the Agilent 2100 Bioanalyzer. RNA samples (100–300 ng) were labeled and amplified using the Ambion Expression Kit. The Affymetrix GeneChip system was used for washing and scanning procedures. The [MoGene-2_0-st] Affymetrix Mouse Gene 2.0 ST Array platform was employed for expression array studies. Microarray signal data normalization across chips utilized the Robust Multichip Analysis (RMA) algorithm (Affymetrix Expression Console v. 1.4.1), as described in our previous study.^12^ The gene expression data for mouse breast tumours is available in the Gene Expression Omnibus (GEO) (GSE252001).

### Polygenic risk score (PGS), mouse tumour susceptibility gene signature score (mTSGSS), and human tumour susceptibility gene signature score (hTSGSS)

Multivariate Cox regression, multivariate linear regression, and multivariate logistic regression were used on significant SNPs from GWAS for the identification of independent and significant SNPs for tumour onset, tumour multiplicity, and tumour metastasis, respectively. The PGS was then constructed as following the formula:

$${PGS}^{phenotype}=\sum_{k=0}^{N^{phenotype}}{Coefficient}_{k}^{phenotype}*\;{SNP}_{k}^{phenotype}$$

where phenotype∊(tumouronset,tumourmultiplicity,tumourmetastasis), $N^{phenotype}$ refers to the number of independent and significant SNPs associated with specific phenotypes, ${Coefficient}_{k}^{phenotype}$ refers to the coefficient of $k^{th}$ SNP associated with a specific phenotype (${SNP}_{k}^{phenotype}$) derived from multivariate analysis. The combination of genes associated with pre-identified SNPs during PGS construction for all three different phenotypes was designated as the mTSGS. For association of mTSGS with responses to docetaxel treatment in 50 genetically diverse F1Bx MMTV-*Erbb2* mice, mTSGS score (mTSGSS) was established using transcriptional levels of the mTSGS, and was defined as follows:

$$\text{mTSGSS}=\sum_{k=0}^{N}{Coefficient}_{k}*\;{Gene\;Expression}_{k}$$

where ${Coefficient}_{k}$ is the coefficient of $k^{th}$ gene derived from multivariate logistic regression.

The corresponding human ortholog of the mTSGS was designated as “hTSGS.” The hTSGS score (hTSGSS) was established in human BC cohorts using the transcriptional levels. Specifically, hTSGSS was defined as follows:

$$\text{hTSGSS}=\sum_{k=0}^{N}{Coefficient}_{k}*\;{Gene\;Expression}_{k}$$

where ${Coefficient}_{k}$ is the coefficient of $k^{th}$ gene derived from multivariate Cox regression analysis in the prognosis study and from multivariate logistic regression analysis in the drug response study.

The risk groups (i.e., low, intermediate, and high) of PGS, mTSGSS, and hTSGSS were defined as the tertiles (top, middle, and bottom) of PGS, mTSGSS and hTSGSS, respectively.

### Human public cohorts

The Cancer Genome Atlas (TCGA) Breast Invasive Carcinoma (TCGA-BRCA) and METABRIC breast cancer transcriptome and clinical data, including PAM50-based molecular subtypes^31^ were downloaded from the cBioPortal (https://www.cbioportal.org/).^32^^,^^33^ The GSE96058 and I-SPY2 (GSE194040) cohorts were downloaded from the Gene Expression Omnibus (GEO) database. The list of genes for human BCs identified in human GWAS was downloaded from the GWAS Catalog database (https://www.ebi.ac.uk/gwas/search?query=rs6928864).^34^ There was no additional modification in the downloaded data during our analyses.

### Statistical analysis

TNMplot (https://tnmplot.com/analysis/) was used to compare gene transcriptional expression in normal and BC tissues based on RNA-seq data.^35^ The difference in overall survival (OS) was assessed by Kaplan–Meier analysis (survminer package in R, version 0.4.8) and log-rank test (survival package in R, version 3.2–3). The p value <0.05 was taken as statistically significant. All data analysis was performed, and plots were generated using R software (version 3.5.0) or IBM SPSS (version 24).

### Ethics

All animal experiments were approved by the Institutional Animal Welfare and Research Committee at Lawrence Berkeley National Laboratory (271004) or the University of Salamanca (PLE2009-0119).

### Role of funders

The funding institutions had no role in the design and conduct of the study; collection, management, analysis, and interpretation of the data; preparation, review, or approval of the manuscript; and decision to submit the manuscript for publication.

## Results

### Variation in mammary tumour onset, multiplicity, and metastasis across CC strains

**Fig. 1:**
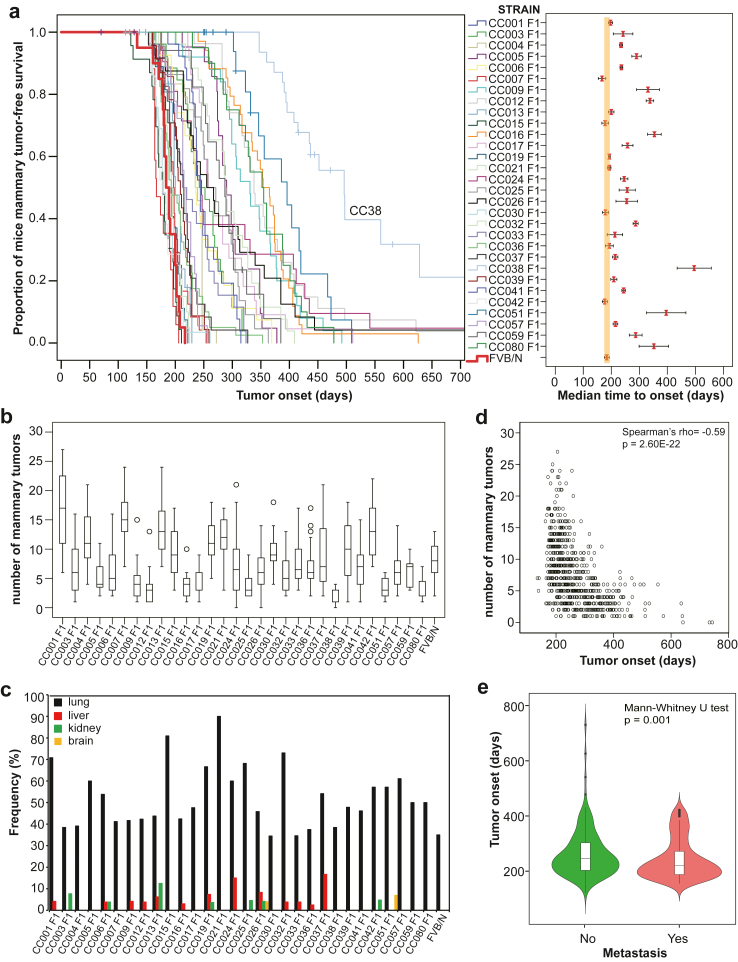
**Variations in *Erbb2*-initiated tumour phenotypes across F1 hybrids of 30 Collaborative Cross (CC) strains and FVB/N MMTV-Erbb2 (CC F1) mice. (a)** Tumour onset of 732 CC F1 of 30 CC strains and 20 FVB/N MMTV-Erbb2 mice. Left panel: The Kaplan–Meier curve for tumour-free survival in each CC F1 strain and FVB/N MMTV-Erbb2 mice. The curve for FVB/N (control) mice was highlighted with a bold red line. Right Panel: median time of tumour onset in each CC F1 strain and FVB/N MMTV-Erbb2 mice. The bars show the 95% confidence interval for median time. **(b)** Multiplicities in the same cohort of mice. Box plot for number of tumours in each CC F1 strain. The low edge of the box represents the lower quartile, while the upper edge of the box represents the upper quartile. The open circles on the diagram show the outliers. **(c)** Frequencies of metastasis in different sites across 30 CC F1 strains. **(d)** The correlation between tumour onset and multiplicities was assessed by the Spearman's rank correlation coefficient and p value. **(e)** The correlation between tumour onset and metastasis was assessed by the Mann–Whitney U test.

A total of 732 female F1 hybrid mice were generated from a cross between FVB/N MMTV-Erbb2 and 30 CC strains and monitored for tumour development over two years. We observed large differences in mammary tumour onset and multiplicity (number of tumours per mouse) across CC strains (Fig. 1a and b; Supplementary Table S1). The median age at tumour onset ranged from 166 to 497 days (Fig. 1a, right panel; Supplementary Table S1). F1 hybrid MMTV-*Erbb2* mice from CC001, CC007, CC013, CC015, CC019, CC021, CC30, CC033, CC036, and CC42 strains had similar onset, while F1 hybrid MMTV-*Erbb2* mice from the remaining CC strains had significantly later onset in comparison to FVB/N MMTV-*Erbb2* mice (Fig. 1a, right panel; Supplementary Table S1). Interestingly, about 20% of CC038 F1 mice did not develop any tumours within the two-year follow-up (Fig. 1a, left panel; Supplementary Table S1). F1 hybrid mice from CC001, CC007, CC013, and CC042 strains developed significantly more tumours, while F1 hybrid mice from CC038, CC080, CC051, and CC012 strains developed significantly less tumours than FVB/N MMTV-*Erbb2* mice (Fig. 1b; Supplementary Table S1). Additionally, we observed a large variation in metastatic incidence across CC strains (Fig. 1c). Although the most frequent metastatic site was the lungs in all strains, we observed an increased frequency of liver metastasis in the CC024 and CC037 strains and an increased frequency of kidney metastasis in the CC013 strain (Fig. 1c; Supplementary Fig. S1 and Supplementary Table S1). We also found that mice with younger age onset developed significantly more tumours in comparison to those with older age onset (p < 0.0001; Spearman's rank correlation; Fig. 1d). Moreover, we found that mice with younger age onset also developed significantly more metastatic tumours (p = 0.01; Mann–Whitney U test; Fig. 1e). These findings indicate that host genetics significantly influences *Erbb2*-driven tumour development and progression.

### Genetic determinants of mammary tumour onset, multiplicity, and metastasis across CC strains

**Fig. 2:**
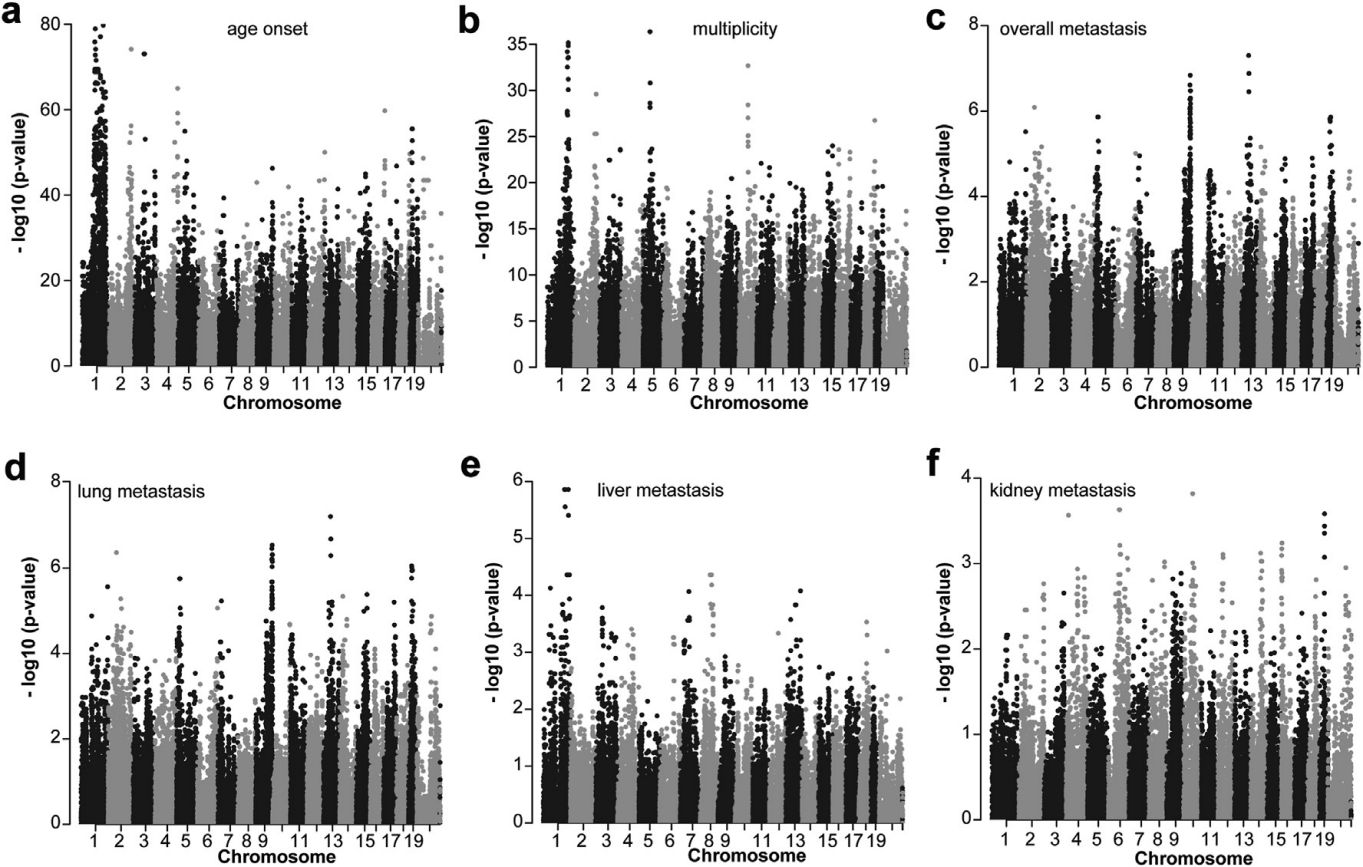
**Genome-wide association study of ErbB2-driven tumour phenotypes in 732 CC F1 mice.** GWAS analysis was performed with 70,273 SNPs located on different chromosomes, including the X chromosome. The Manhattan plot for **(a)** tumour onset, where the p value was obtained by the Kaplan–Meier method and log-rank test at each SNP; **(b)** tumour multiplicities, where the p value was generated by the Mann–Whitney U test at each SNP; **(c)** overall metastasis (metastasis in any sites); **(d)** lung metastasis; **(e)** liver metastasis; and **(f)** kidney metastasis. For all metastatic phenotypes, the p value was calculated by the Chi-square test at each SNP. The figure plots the –log_10_ (p value) on the y-axis *versus* the chromosome position on the x-axis for each SNP, and each dot in the figure represents one SNP.

To investigate the contribution of genetic variants to mammary tumour onset, tumour multiplicities, and metastasis, GWAS analysis was performed with 70,273 SNPs across 30 CC F1 strains. We identified 1525 SNPs significantly associated with tumour onset (p < 1.00E-30; log-rank test) corresponding to 275 known genes (Fig. 2a; Supplementary Fig. S2a, Supplementary Tables S2 and S3), 800 SNPs significantly associated with the number of tumours (p < 1.00E-15; Mann–Whitney U test) corresponding to 194 known genes (Fig. 2b; Supplementary Fig. S2b, Supplementary Tables S2 and S3), 588 SNPs significantly associated with overall tumour metastasis (p < 1.00E-4; Chi-square test) corresponding to 171 known genes (Fig. 2c; Supplementary Fig. S2c, Supplementary Tables S2 and S3), 568 SNPs significantly associated with lung metastasis (p < 1.00E-4; Chi-square test) corresponding to 168 known genes (Fig. 2d; Supplementary Tables S2 and S3) and 23 SNPs significantly associated with liver metastasis (p < 1.00E-4; Chi-square test) corresponding to 12 known genes (Fig. 2e; Supplementary Tables S2 and S3). We did not find any SNPs significantly associated with kidney metastasis (Fig. 2f; Supplementary Table S2). Given the limited number of mice with kidney metastasis, our study may have lacked the statistical power to detect SNPs.

To elucidate the mechanisms underlying tumour susceptibility, we used WebGestalt^30^ to evaluate functional enrichment analysis of candidate susceptibility genes for each phenotype using the Kyoto Encyclopedia of Genes and Genomes (KEGG) pathway. For tumour onset, the genes were predominantly enriched in pathways such as Ras (p = 0.0033; hypergeometric test), Hedgehog signaling (p = 0.0061; hypergeometric test), and ECM-receptor interaction (p = 0.0091; hypergeometric test), among others (Supplementary Fig. S3a). In the context of tumour multiplicity, there was significant enrichment in pathways like ECM-receptor interaction (p = 0.0087; hypergeometric test) and transcriptional misregulation in cancer (p = 0.010; hypergeometric test) (Supplementary Fig. S3b). For metastasis, pathways such as gap junction (p = 0.0078; hypergeometric test) and regulation of lipolysis in adipocytes (p = 0.0013; hypergeometric test) were predominantly represented (Supplementary Fig. S3c).

### Establishment of polygenic risk scores for tumour onset, multiplicity, and metastasis

**Fig. 3:**
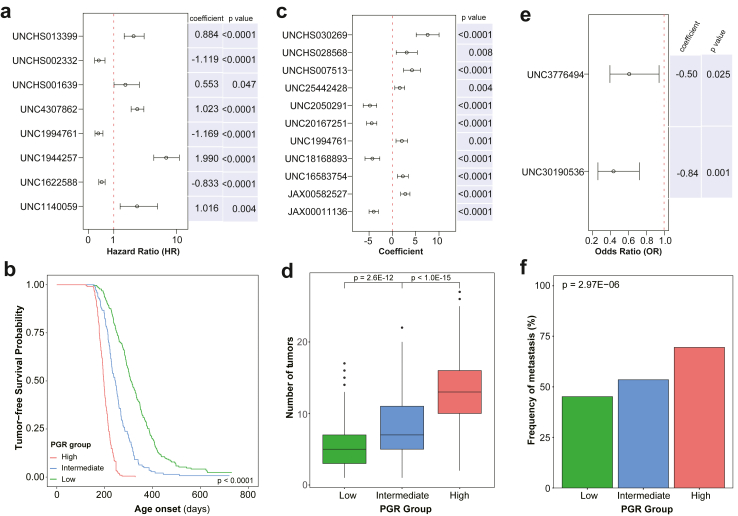
**Polygenic risk score for ErbB2-driven tumour phenotypes in 732 CC F1 mice. (a)** SNPs and their corresponding coefficients for creating polygenic risk score for tumour onset based on multivariate Cox analysis. The coefficients and p values were obtained from multivariate Cox regression (**b**) The polygenic risk score for tumour onset. The Kaplan–Meier curve for tumour-free survival among different polygenic risk groups. The p value was obtained from the log-rank test. (**c**) SNPs and their corresponding coefficients for creating polygenic risk score for tumour multiplicities. The coefficients and p values were obtained from multivariate linear regression. **(d)** The polygenic risk score for tumour multiplicities. Box plot for number of tumours among different polygenic risk groups (PGR). The p values were obtained from the Mann–Whitney U test. (**e**) SNPs and their corresponding coefficients for creating polygenic risk score for overall tumour metastasis. The coefficients and p values were obtained from multivariate logistic regression. **(f)** The polygenic risk score for overall metastasis. Frequencies of metastasis among different polygenic risk groups. The p value was obtained from the Chi-square test.

We used multivariate analysis to determine the most critical SNPs for each phenotype. Multivariate Cox regression analysis identified SNPs in 8 genes (*Stx6, Ramp1, Traf3ip1, Nckap5, Pfkfb2, Trmt1l, Rprd1b,* and *Rer1*) that were independently associated with age of tumour onset (Fig. 3a). The polygenic risk (PGR) score of SNPs in these eight genes was significantly associated with age of tumour onset (Fig. 3b). Multivariate linear regression analysis identified SNPs in 11 genes (*Sepsecs, Rhobtb1, Tsen15, Abcc3, Arid5b, Tnr, Dock2, Tti1, Fam81a, Stx6,* and *Oxr1*) that were independently associated with the tumour multiplicities (Fig. 3c). The PGR score of SNPs in these 11 genes was significantly associated with the number of tumours (Fig. 3d). Multivariate logistic regression analysis identified SNPs in 2 genes (*Plxna2* and *Tbc1d31*) that were independently associated with tumour metastasis (Fig. 3e). The PGR score of SNPs in these two genes was significantly associated with tumour metastasis (Fig. 3f). We pooled all 20 genes (*Stx6, Ramp1, Traf3ip1, Nckap5, Pfkfb2, Trmt1l, Rprd1b, Rer1, Sepsecs, Rhobtb1, Tsen15, Abcc3, Arid5b, Tnr, Dock2, Tti1, Fam81a, Oxr1, Plxna2* and *Tbc1d31*) together as the mouse tumour susceptibility gene signature (mTSGS). Additionally, we conducted RNA-seq analysis of 60 CC F1 tumours and found that based on transcriptional levels of 20 genes, CC F1 mice were clustered into two groups that differ in tumour onset, multiplicity, and metastasis by unsupervised clustering analysis (Supplementary Fig. S4), suggesting that transcriptional levels of 20 genes are associated with individual mouse tumour susceptibility.

### hTSGS score (hTSGSS) is significantly associated with the prognosis of human breast cancer

**Fig. 4:**
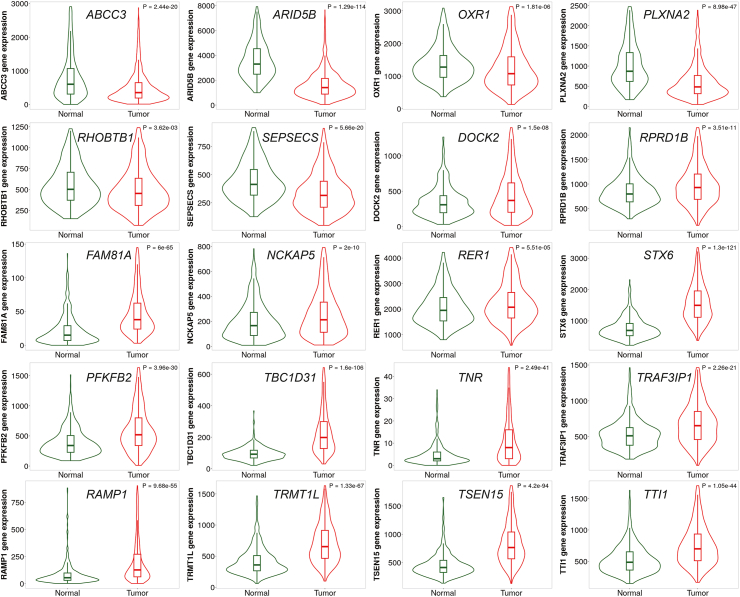
**The human orthologs of mTSGS are transcriptionally altered in human breast cancers.** The violin plots were generated, and p values were obtained from the Mann–Whitney U test between normal and cancer tissues using TNMplot.

**Fig. 5:**
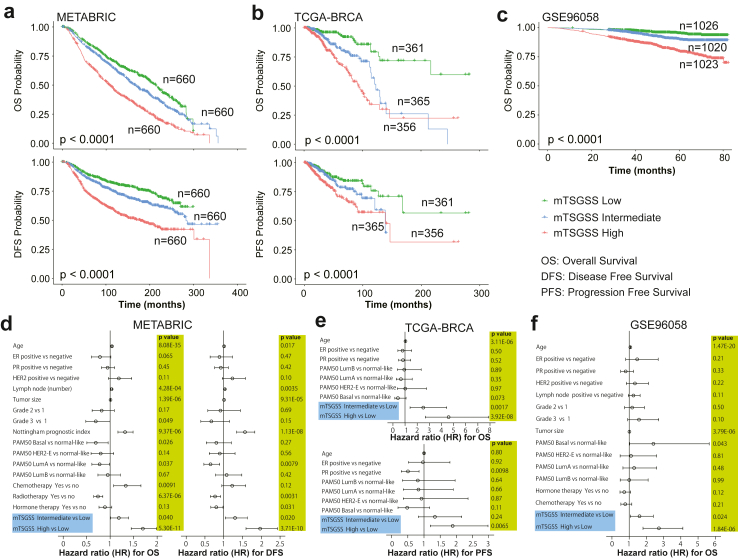
**Association of the hTSGSS with prognosis in human breast cancer.** hTSGSS was created based on transcriptional expression by multivariate Cox regression. The patients were divided into three groups based on hTSGSS (top, intermediate, and bottom tertile). hTSGSS was a significant and independent prognostic factor. **(a)** Kaplan–Meier survival curves for disease-free survival (DFS) (bottom panel) and overall survival (OS) (top panel) are presented in the METABRIC dataset. **(b)** Kaplan–Meier survival curves for progression-free survival (PFS) (bottom panel) and OS (top panel) in the TCGA-BRCA dataset. **(c)** Kaplan–Meier survival curves for OS in the GSE96058 dataset. The p values shown were obtained from the log-rank test. **(d)** The forest plot shows the results of the multivariate Cox regression model for exploring clinical factors, PAM50, and mTSGSS for OS (left panel) and DFS (right panel) in the METABRIC dataset. **(e)** The forest plot shows the results of the multivariate Cox regression model for exploring clinical factors, PAM50 and mTSGSS for OS (top panel) and PFS (bottom panel) in the TCGA-BRCA dataset. **(f)** The forest plot shows the results of the multivariate Cox regression model for exploring clinical factors, PAM50, and mTSGSS for OS in the GSE96058 dataset. The bars show the 95% confidence interval for the hazard ratio. The hazard ratios and p values were obtained from multivariate Cox regression.

To evaluate the importance and clinical value of mTSGS in human breast cancer (BC), we identified human orthologs of mTSGS named hTSGS, then used TNMplot to examine their transcriptional expression in BC and found that all genes transcriptionally altered. The expression levels of *ABCC3*, *ARD5B*, *OXR1*, *PLXNA2*, *RHOBTB1,* and *SEPSECS* gene were significantly reduced, while the expression levels of the remaining genes were significantly elevated in comparison to the normal mammary tissues (Fig. 4). Additionally, we demonstrated that individual PGR gene sets related to tumour onset, multiplicity, and metastasis had significantly prognostic value (Supplementary Fig. S5a–i); and combining them as one signature had significantly more predictive power than individual sets alone, as evidenced by Cindex (p < 0.0001; Mann–Whitney U test; Supplementary Fig. 5j–l). Therefore, we established a hTSGS score (hTSGSS) based on the transcriptional levels of combined genes, i.e., hTSGS (details in the method). We found that hTSGSS was significantly associated with different clinical outcomes, such as overall survival (OS), disease-free survival (DFS), and progression-free survival (PFS) in multiple human BC datasets (Fig. 5a–c; Supplementary Tables S4–S6). Patients with low mTSGSS have a favourable prognosis (Fig. 5a–c). Moreover, hTSGSS was consistently associated with these clinical outcomes in each PAM50 molecular subtype (Supplementary Fig. S6). Finally, using multivariate Cox regression analyses (including clinical factors, PAM50 molecular subtype, hTSGSS), we demonstrated that the prognostic impact of hTSGSS is independent of clinical factors (such as age, ER, and PR) and PAM50 molecular subtypes (Fig. 5d–f).

### hTSGSS predicts responses to different treatments in human breast cancer

**Fig. 6:**
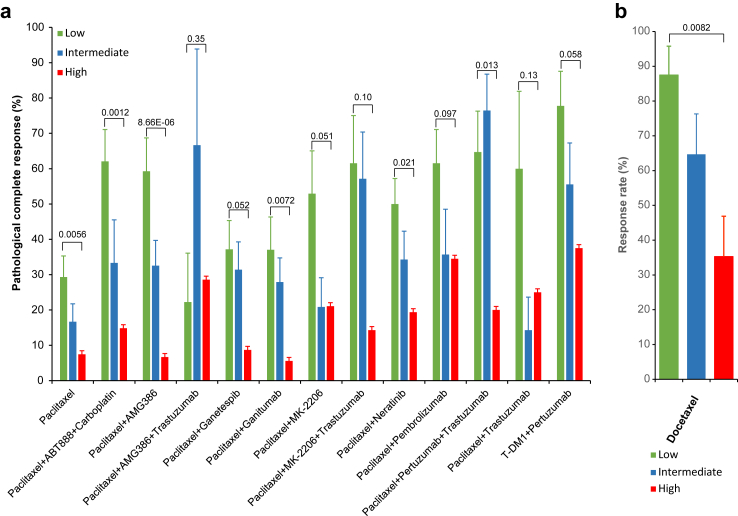
**hTSGSS is a predictor for response to different treatment regimens in human breast cancer. (a)** hTSGSS significantly correlated with pathological complete response (pCR) to different treatment regiments in the human I-SPY2 dataset (GSE194040). **(b)** mTSGSS significantly correlated with response to docetaxel treatment in genetically diverse F1Bx MMTV-ErbB2 mice. The p values were obtained from the Chi-square test.

Using the I-SPY2 dataset (GSE194040) that contains a total of 987 patients from 13 arms of the neoadjuvant treatment trial,^36^ we discovered that hTSGSS is significantly correlated with pathological complete response (pCR) to different treatment regiments (Fig. 6a; Supplementary Table S7). Overall, patients with low hTSGSS have a higher pCR rate in comparison to those with high hTSGSS for 6 of 13 treatment regimens (Fig. 6a).

Taxanes are still highly active chemotherapy agents used in metastatic BC. To evaluate the predictive value of mTSGSS for the responses to taxane, 50 genetically diverse F1Bx MMTV-*Erbb2* mice were treated with docetaxel when the tumour volume reached 500 mm^3^, and the treatment responses for each mouse were assessed (detail see method and material section). As we found in human studies, mTSGSS generated from the transcript levels measured in the pre-treatment biopsy was able to predict the response to docetaxel treatment, and the tumours with low mTSGSS were more likely to respond to docetaxel treatment (Fig. 6b).

**Fig. 7:**
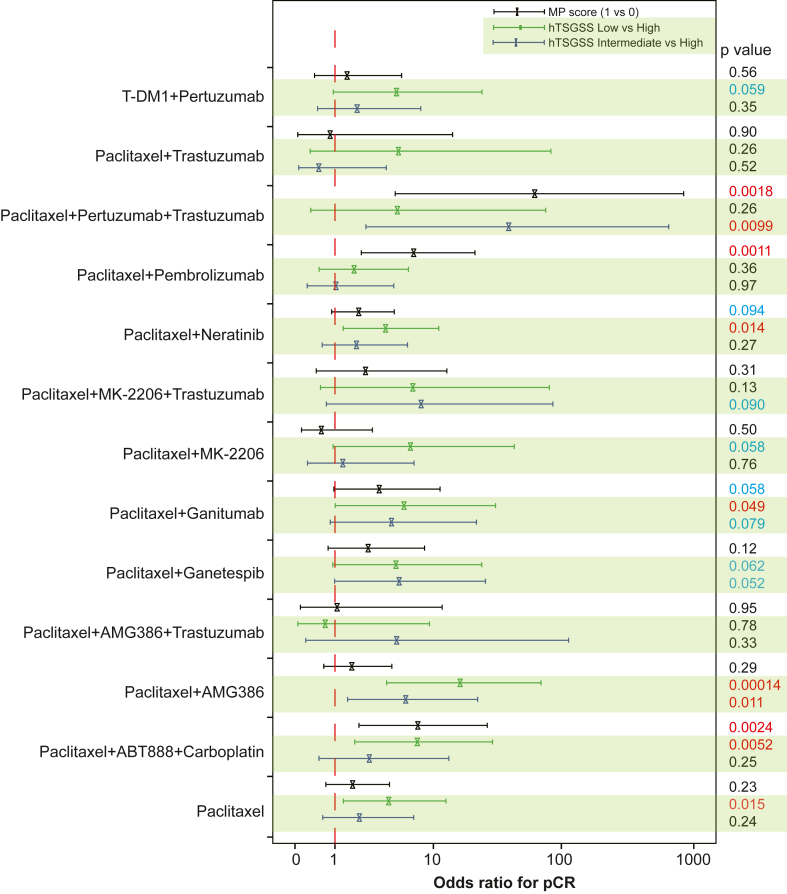
**The predictive value of hTSGSS for response to different treatment regimens is independent of MammaPrint in human breast cancer.** The forest plot shows the results of the multivariate logistic regression model including hTSGSS and MammaPrint for pCR. The bars show the 95% confidence interval for the odds ratio. The odds ratios and p values were obtained from multivariate logistic regression.

Finally, multivariate logistic regression analysis showed that the predictive value of hTSGSS in pCR is independent of the MammaPrint (MP) score (Fig. 7). These findings indicate that the hTSGSS is equal to or better than MP in all treatment groups except those containing pembrolizumab.

## Discussion

Even with substantial progress in ERBB2-targeted therapies, resistance–whether acquired or intrinsic–remains a formidable challenge. This resistance is thought to arise from a range of mechanisms, including the activation of alternative signaling pathways, ERBB2 gene mutations, and tumour heterogeneity.^37^^,^^38^ Given these challenges, identifying patients who stand to benefit the most from a particular treatment is imperative, as this enhances therapeutic efficacy and reduces potential toxicity. Therefore, a better understanding of the biology of ERBB2-driven cancer supports the development of new treatment options for patients. The goal of this study was to identify genetic factors that control Erbb2-driven mammary tumour development and metastasis using a large cohort of genetically diverse CC mice. Our findings demonstrate that there is a large strain-dependent variation in *Erbb2*-initiated tumourigenic phenotypes, and analysis of such variability in CC mice can reveal the underlying genetic basis in human BCs.

This study confirmed the significance of many loci that were previously identified using the F1 backcross approach,^12^ but as expected, because of the increased genetic divergence in the CC mice, we identified many additional genetic loci that were strongly associated with tumourigenic phenotypes. Using all tumour phenotypes, we discovered a total of 551 candidate genes, human orthologs for which are shown in Supplementary Table S8. Twenty-three of these genes (*RTKN2*, *PHF20*, *CPEB3*, *BCL2*, *NIPSNAP1*, *TENM2*, *PBX1*, *ITPR2*, *WWOX*, *HORMAD2*, *DNM3*, *PTPRN2*, *PRKG1*, *IQCA1*, *GPR161*, *SORCS3*, *PCM1*, *EBF2*, *JMJD1C*, *TGFBR2*, *SLC39A11*, *SEC14L4,* and *NYAP2*) have been found by human GWAS for BCs based on the GWAS Catalog database.^34^ There are 87 overlapping susceptibility genes between tumour onset and multiplicity, but only seven overlapping susceptibility genes between onset and overall tumour metastasis, and only five overlapping susceptibility genes between multiplicity and overall tumour metastasis (Supplementary Fig. S7). Only two susceptibility genes (*Nckap5* and *Ptprt*) overlap among all tumour phenotypes (onset, multiplicity, and metastasis) (Supplementary Fig. S7). *PTPRT*, a member of the protein tyrosine phosphatase (PTP) family, has been reported to be a tumour suppressor gene in BC and other cancers.^39^, ^40^, ^41^, ^42^, ^43^, ^44^, ^45^ *NCKAP5*, potentially functioning in microtubule bundle formation and microtubule depolymerization, is less studied, and polymorphisms in this gene are reported to be associated with the clinical outcome of patients with gastric cancer in a recent study.^46^ Overall, our study suggests different genetic factors controlling tumour onset, multiplicity, metastasis, and the site of metastasis.

In the contemporary landscape of personalized medicine, the identification of biomarkers capable of forecasting treatment responses is of paramount importance. These predictive markers further tailor therapeutic interventions, circumventing unneeded drug exposure in patients unlikely to experience clinical advantages. As our comprehension of the molecular underpinnings of ERBB2-positive breast cancer expands, new avenues will open for treatments that are even more patient-specific. Of note, genomic tests, especially those centered on gene expression signatures, are becoming increasingly prominent.^47^^,^^48^ In this study, we identified a mouse tumour susceptibility gene signature (mTSGS) comprised of 20 genes (*Stx6, Ramp1, Traf3ip1, Nckap5, Pfkfb2, Trmt1l, Rprd1b, Rer1, Sepsecs, Rhobtb1, Tsen15, Abcc3, Arid5b, Tnr, Dock2, Tti1, Fam81a, Oxr1, Plxna2,* and *Tbc1d31*), and showed that transcriptional expression of hTSGS in human BC can be used to predict prognosis and response to different cancer treatments in patients. Moreover, we demonstrated that our signature stands as a prognostic indicator, distinct from other recognized signatures like the PAM50 molecular subtype^31^ and MammaPrint.^49^ The integration of our signature with different BC treatment regimens might enhance the precision of adjuvant treatment decisions for patients with BC. Our study further indicates that the CC mouse model can serve as an invaluable pre-clinical model for genetic understanding of drug resistance.

A major strength of this study is the use of a very large scale genetic study to identify many new genetic loci contributing to ERBB2-driven cancers. Further, we used multipronged approach to evaluate clinical relevance of the mouse tumour susceptibility gene signature for patients with BC, which ultimately provide personalized prevention and customized treatments. However, we awakened there were some limitations. Further biological and functional studies of many new candidate genes discovered in this study are required to substantially enhance our understanding of ERBB2 biology and to develop new treatment approaches for ERBB2+ BCs. Moreover, the clinical utility of hTSGSS needs to be further validated in prospective cohort studies to confirm its predictive power in facilitating more personalized therapy in patients with BC.

In conclusion, we have identified many new susceptibility genes for ERBB2-driven cancer. Translational studies indicate that hTSGSS may serve as a biomarker for tailoring treatment to patients with BC.

## Contributors

JHM, HC, and JPL conceived and designed the overall study; JHM and JPL acquired funding; HY, HC, and JHM performed the experimental study in a large cohort of CC mice; LH, XW, and JLI made some contributions to the CC study; ABG, NGS, RCC, AJN, MJPB, and JPL performed mouse therapy study; PW, HC, and JHM performed data analysis; JLI, PW, AMS, HC, JPL, and JHM were involved in interpretation of results; JHM, JPL and HC wrote the manuscript; DWT and AB provided suggestions and made substantial manuscript editing; all authors have read, edited and approved the final version of the manuscript. JHM, HC, and JPL have verified the underlying data.

## Data sharing statement

The gene expression data for mouse breast tumours is available in the Gene Expression Omnibus (GEO) (GSE252001) and the National Center for Biotechnology Information (NCBI) BioProject Repository (https://www.ncbi.nlm.nih.gov/bioproject) under the BioProject “PRJNA1122577”.

The human datasets in this study are publicly available. The Cancer Genome Atlas (TCGA) (TCGA-BRCA) and METABRIC breast cancer transcriptome and clinical data, including PAM50-based molecular subtypes^31^ were downloaded from the cBioPortal (https://www.cbioportal.org/).^32^^,^^33^ The GSE96058 and I-SPY2 (GSE194040) cohorts were downloaded from the Gene Expression Omnibus (GEO) database. The list of genes for human BCs identified in human GWAS was downloaded from the GWAS Catalog database (https://www.ebi.ac.uk/gwas/search?query=rs6928864).^34^

## Declaration of interests

The authors declare no competing interests.

## Appendix A Supplementary data

Supplementary Figures Tables Final
