# Supplementary material for: A susceptibility gene signature for ERBB2-driven mammary tumour development and metastasis in collaborative cross mice
Source: eBioMedicine. 2024 Jul 26;106:105260. doi: 10.1016/j.ebiom.2024.105260 (PMC11338061; doi:10.1016/j.ebiom.2024.105260)
Supplement: Supplementary Figures Tables Final [file mmc1.pdf]

Supplementary Figures and Tables

A susceptibility gene signature for ERBB2-driven mammary tumor development and metastasis in Collaborative Cross mice with human translational value

Hui Yang[1,2], Xinzhi Wang[1,3], Adrián Blanco-Gómez[4,5], Li He[1,6], Natalia García-Sancha[4,5], Roberto Corchado-Cobos[4,5], Manuel Jesús Pérez-Baena[4,5], Alejandro Jiménez-Navas[4,5], Pin Wang[1,7], Jamie L Inman[1], Antoine M Snijders[1,8], David W Threadgill[9,10], Allan Balmain[11], Hang Chang[1,8]*, Jesus Perez-Losada[4,5]*, Jian-Hua Mao[1,8]*

Supplementary Figures

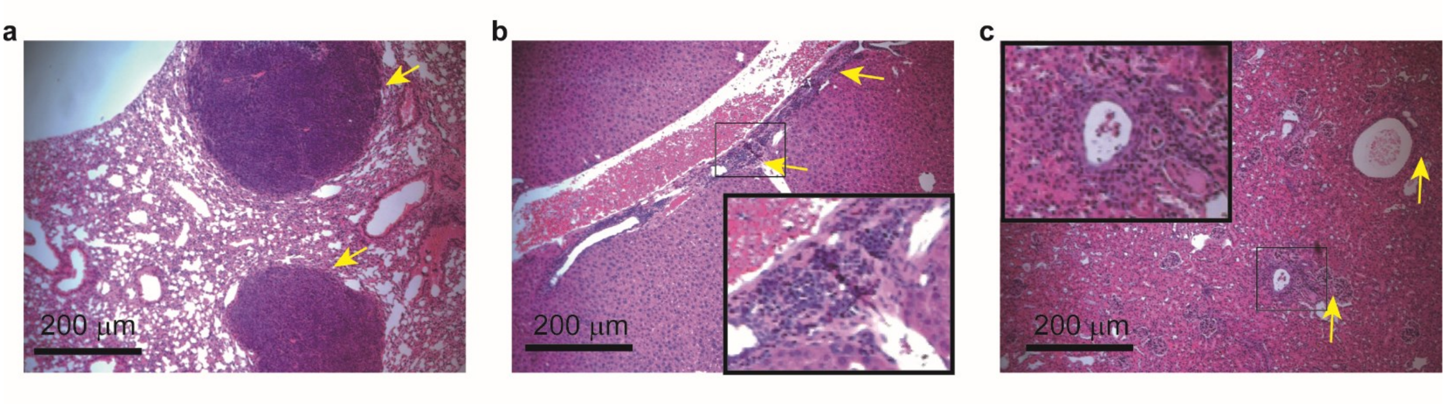

Supplementary Figure S1. Representative histological image for metastasis. (**a**) Metastasis in lung. (**b**) metastasis in the liver. (**c**) Metastasis in the kidney. The yellow arrows point to metastatic foci.

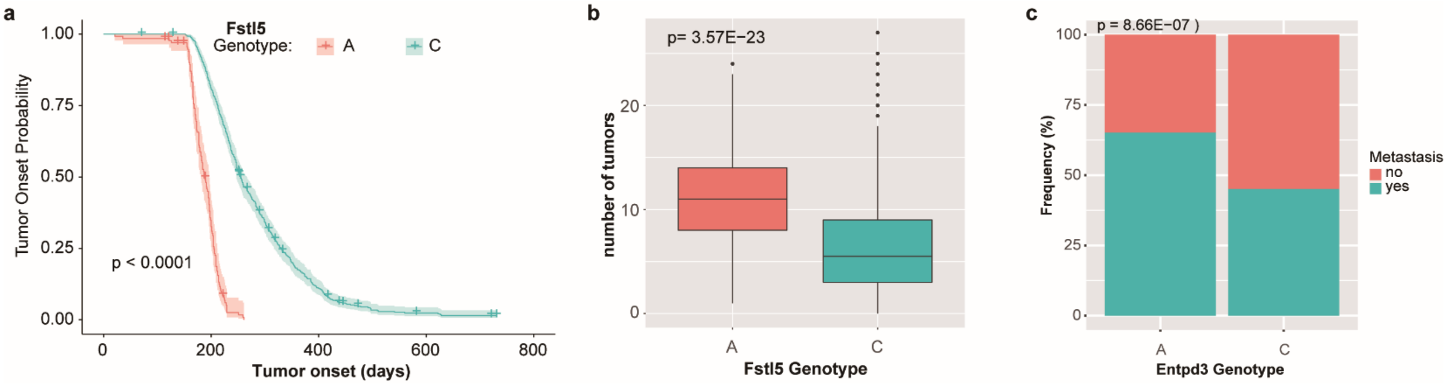

Supplementary Figure S2. Association of representative SNPs with *Erbb2*-driven tumor phenotype. (**a**) Significant association of SNP in *Fstl5* gene with tumor onset. The p value was obtained from the Kaplan-Meier method and the log-rank test. (**b**) Significant association of SNP *Fstl5* gene with tumor multiplicities. The p value was obtained from the Mann-Whitney U test. (**c**) Significant association of SNP in *Entpd3* gene with overall tumor metastasis. The p value was obtained from the Chi-square test.

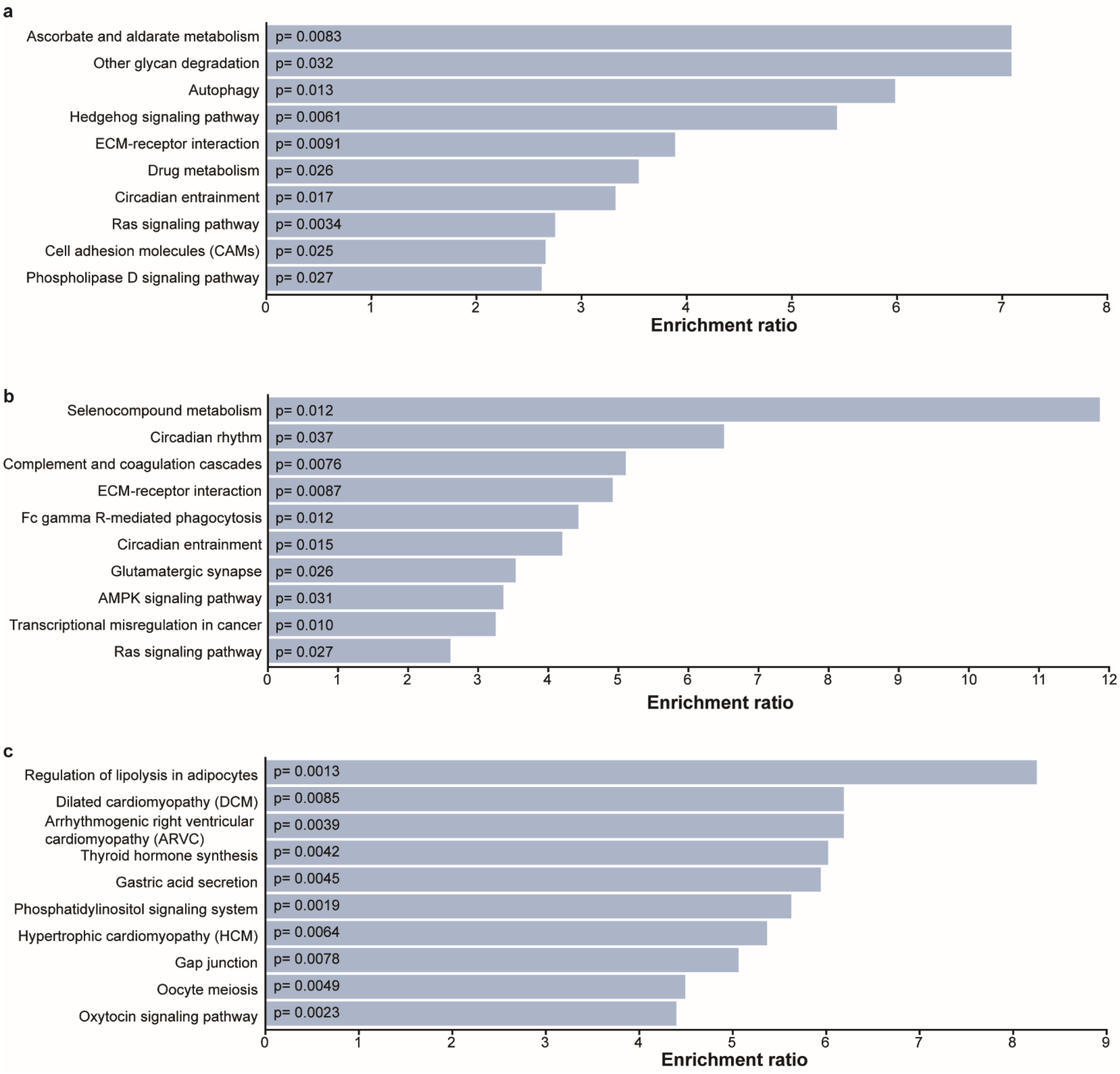

Supplementary Figure S3. KEGG pathway enrichment analysis of candidate genes for ErbB2-driven tumor phenotypes. (a) Tumor onset. **(b)** Tumor multiplicities. **(c)** Overall metastasis. The p values are obtained from hypergeometric test.

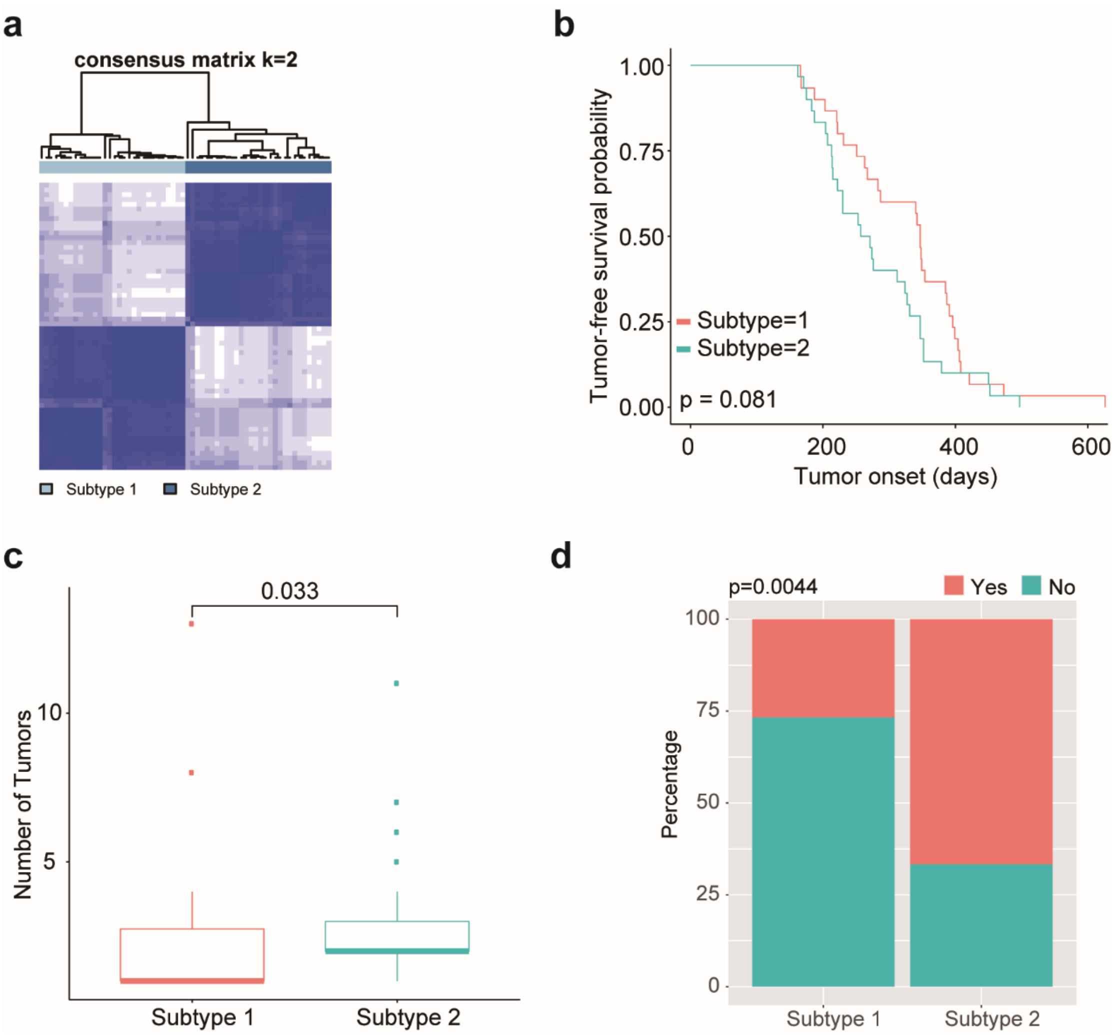

Supplementary Figure S4. Subtyping mouse mammary tumors based on transcriptional level of 20 mTSGS genes. (**a**) Consensus clustering model for mouse mammary tumor subtypes discovery and inference. (**b-d**) Subtypes show significant differences in tumor onset (**b**), multiplicity (**c**), and metastasis (**d**). The p values were obtained from log-rank test (**b**), Mann-Whitney U test (**c**) and Chi-square test (**d**).

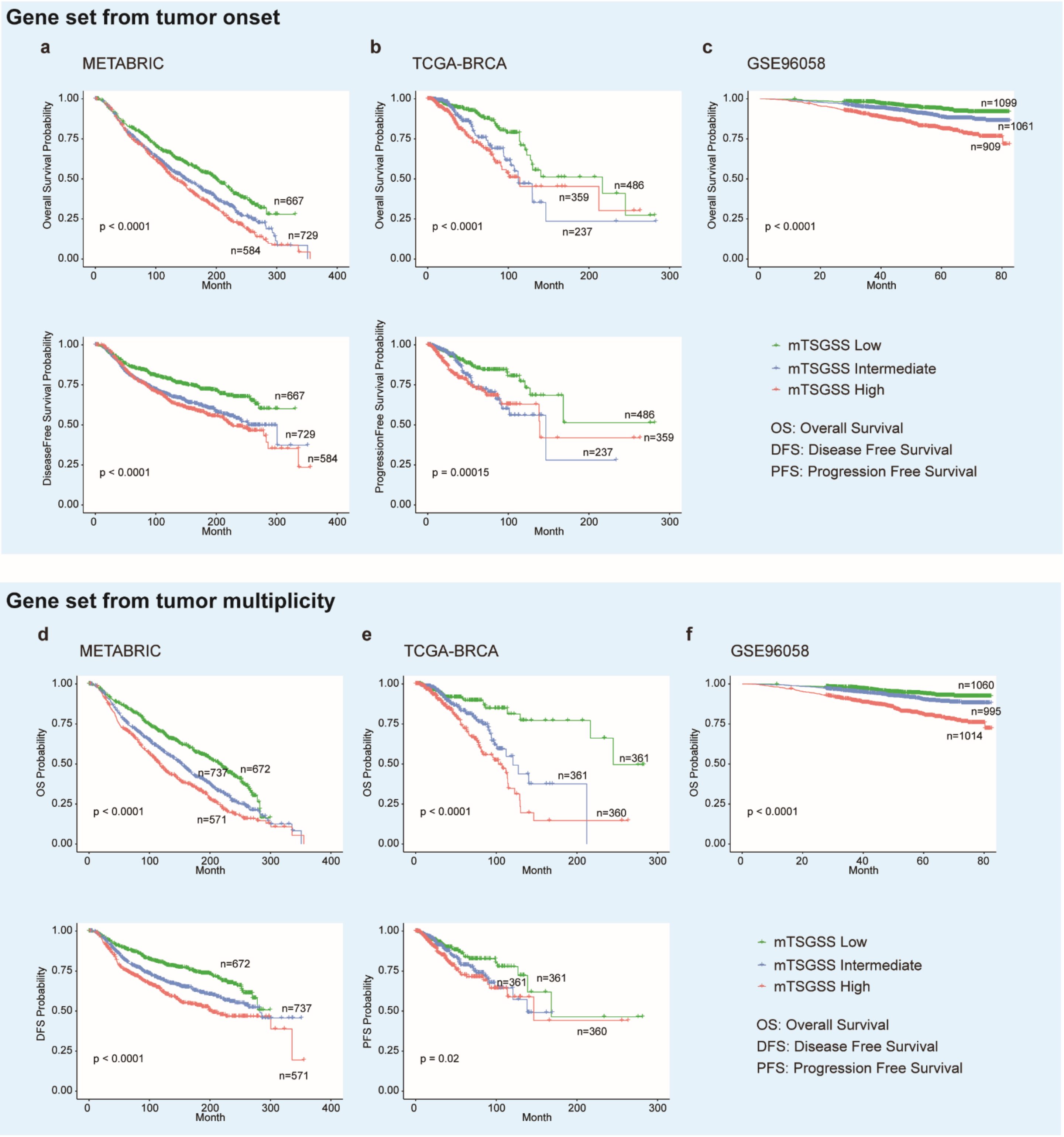

Supplementary Figure S5. Prognostic value of individual PGR gene sets in human breast cancer. Tumor onset PGR gene set (a-c): (a) Kaplan-Meier survival curves for disease-free (DFS) and overall (OS) survival are presented in the METABRIC dataset. **(b)** Kaplan-Meier survival curves for progression-free survival (PFS) and OS in TCGA-BRCA dataset. **(c)** Kaplan-Meier survival curves for OS in the GSE96058 dataset. **Tumor multiplicity gene set (d-f): (d)** Kaplan-Meier survival curves for DFS and OS are presented in the METABRIC dataset. **(e)** Kaplan-Meier survival curves for PFS and OS in TCGA-BRCA dataset. **(f)** Kaplan-Meier survival curves for overall OS in the GSE96058 dataset. The p values shown were obtained from the log-rank test.

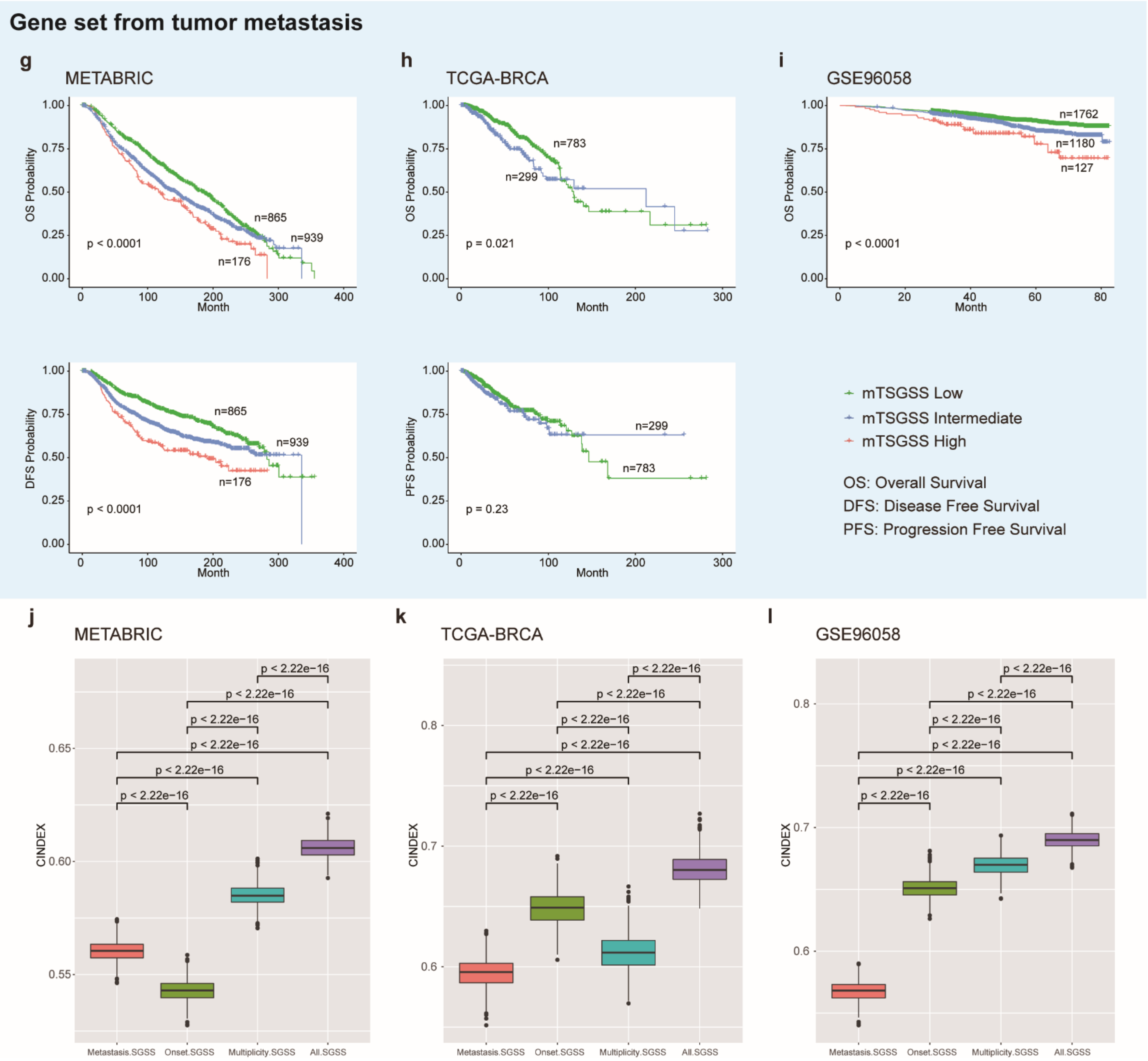

Supplementary Figure S5. (continued) Tumor metastasis gene set (g-i): (g) Kaplan-Meier survival curves for DFS and OS are presented in the METABRIC dataset. **(h)** Kaplan-Meier survival curves for PFS and OS in TCGA-BRCA dataset. **(i)** Kaplan-Meier survival curves for overall OS in the GSE96058 dataset. The p values shown were obtained from the log-rank test. (**j-l**) Comparing predictive power between different PGR gene sets and combinations of them by Cindex in METARBRIC (**j**), TCGA-BRCA (**k**), and GSE96058 (**l**) cohorts. The p values were obtained from the Mann-Whitney U test.

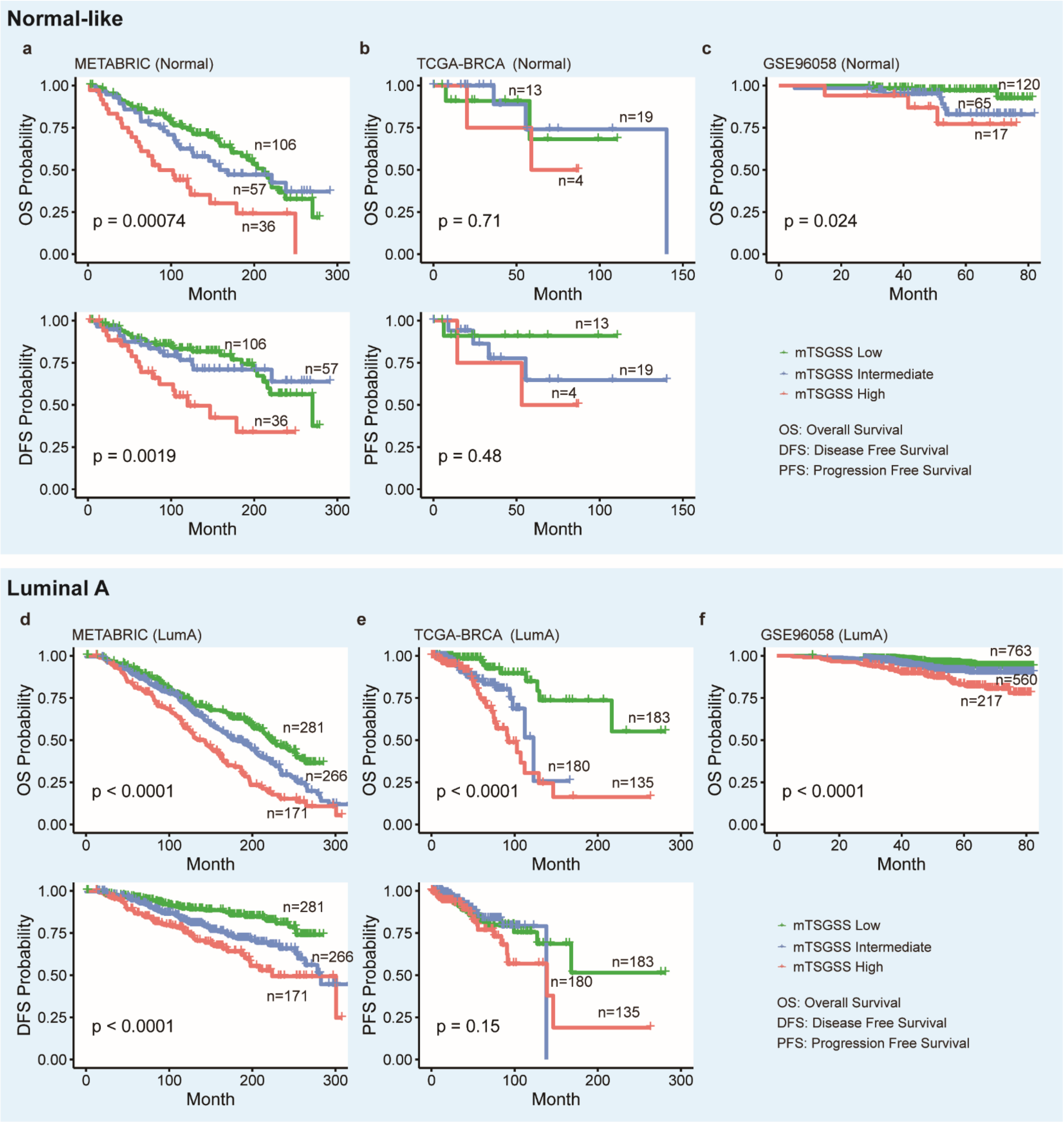

Supplementary Figure S6. Association of hTSGSS with clinical outcomes in each PAM50 molecular subtype. Normal-like (a-c): (a) Kaplan-Meier survival curves for disease-free (DFS) and overall (OS) survival are presented in the METABRIC dataset. **(b)** Kaplan-Meier survival curves for progression-free survival (PFS) and OS in TCGA-BRCA dataset. **(c)** Kaplan-Meier survival curves for OS in the GSE96058 dataset. **Luminal A (d-f): (d)** Kaplan-Meier survival curves for DFS and OS are presented in the METABRIC dataset. **(e)** Kaplan-Meier survival curves for PFS and OS in TCGA-BRCA dataset. **(f)** Kaplan-Meier survival curves for overall OS in the GSE96058 dataset. The p values shown were obtained from the log-rank test.

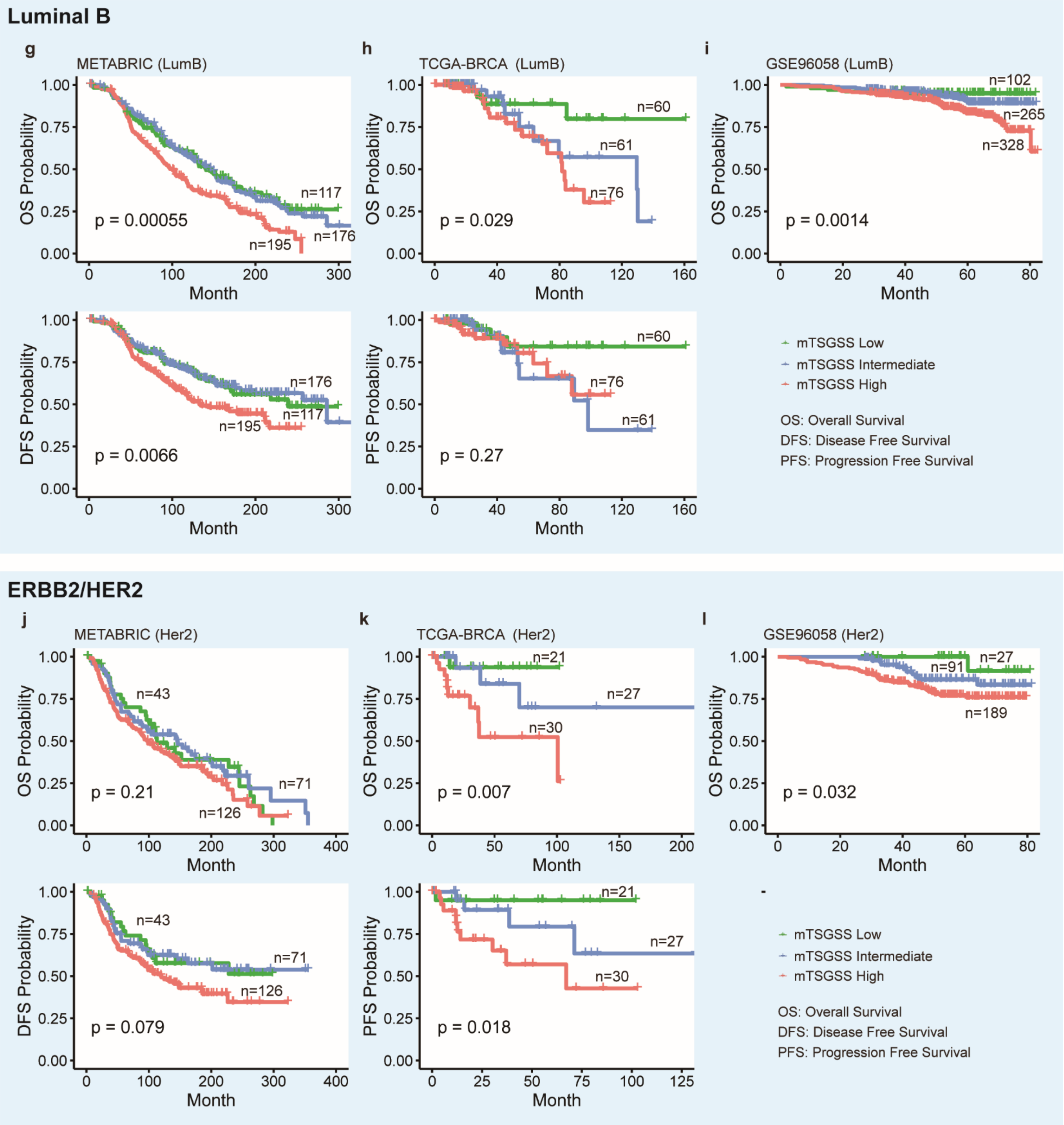

Supplementary Figure S6. (continued). Luminal B (g-i): (g) Kaplan-Meier survival curves for DFS and OS are presented in the METABRIC dataset. **(h)** Kaplan-Meier survival curves for PFS and OS in TCGA-BRCA dataset. **(i)** Kaplan-Meier survival curves for OS in the GSE96058 dataset. **ERBB2/HER2 (j-l): (j)** Kaplan-Meier survival curves for DFS and OS are presented in the METABRIC dataset. **(k)** Kaplan-Meier survival curves for PFS and OS in TCGA-BRCA dataset. **(l)** Kaplan-Meier survival curves for OS in the GSE96058 dataset. The p values shown were obtained from the log-rank test.

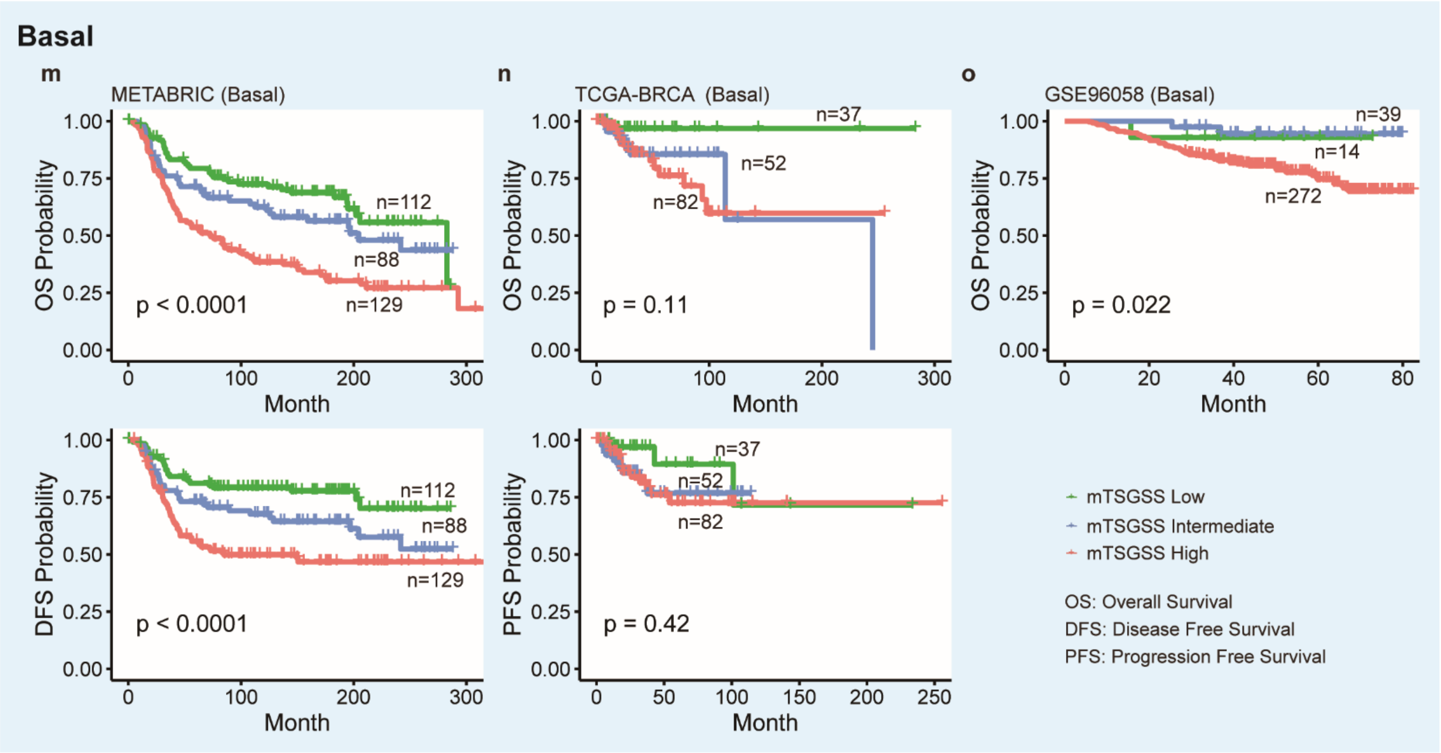

Supplementary Figure S6. (continued). Basal (m-o): (m) Kaplan-Meier survival curves for DFS and OS are presented in the METABRIC dataset. **(n)** Kaplan-Meier survival curves for PFS and OS in TCGA-BRCA dataset. **(o)** Kaplan-Meier survival curves for OS in the GSE96058 dataset. The p values shown were obtained from the log-rank test.

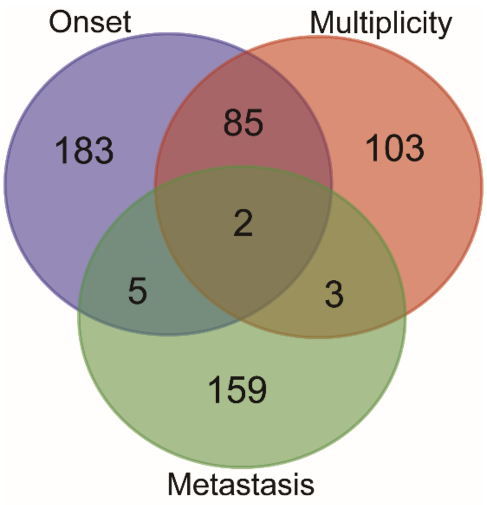

Supplementary Figure S7. Venn diagram of the number of candidate genes among different *Erbb2*-driven tumor phenotypes.

Supplementary Tables

Supplementary Table S1. Summary of *Erbb2*-driven tumor phenotypes in each CC mouse strain and FVB/N mice.

strain	Number of mice	Tumor onset			Multiplicity		Metastasis			
		median (days)	95% CI for median		median	range	overall	lung	liver	kidney
			Lower	Upper						
CC001	24	197.00	189.80	204.20	17	6~27	75.0%	70.8%	4.2%	0.0%
CC003	26	242.00	207.02	276.98	6	1~16	38.5%	38.5%	0.0%	7.7%
CC004	24	235.00	229.46	240.54	11	4~21	39.1%	39.1%	0.0%	0.0%
CC005	23	290.00	273.18	306.82	4	2~11	60.0%	60.0%	0.0%	0.0%
CC006	27	236.00	230.91	241.09	5	2~16	46.2%	46.2%	3.8%	3.8%
CC007	17	167.00	153.55	180.45	15	7~24	41.2%	41.2%	0.0%	0.0%
CC009	25	331.00	290.20	371.80	4	1~15	41.7%	37.5%	4.2%	0.0%
CC012	27	338.00	324.43	351.57	3	1~13	42.3%	42.3%	3.8%	0.0%
CC013	22	200.00	190.43	209.57	13	7~24	43.8%	43.8%	6.3%	12.5%
CC015	23	178.00	166.26	189.74	9	3~17	81.0%	81.0%	0.0%	0.0%
CC016	34	354.00	329.71	378.29	4	1~10	45.5%	42.4%	3.0%	0.0%
CC017	22	258.00	238.46	277.54	3	1~9	47.6%	47.6%	0.0%	0.0%
CC019	27	193.00	189.61	196.39	11	5~18	66.7%	66.7%	7.4%	0.0%
CC021	20	193.00	187.16	198.84	12	3~17	90.0%	90.0%	0.0%	0.0%
CC024	21	246.00	232.54	259.46	7	0~21	65.0%	60.0%	15.0%	0.0%
CC025	22	257.00	228.27	285.73	3	2~9	68.2%	68.2%	0.0%	4.5%
CC026	24	255.00	216.59	293.41	6	0~14	45.8%	45.8%	4.2%	4.2%
CC030	29	179.00	168.45	189.55	9	4~18	37.9%	34.5%	3.4%	0.0%
CC032	26	287.00	278.01	295.99	5	2~13	73.1%	73.1%	3.8%	0.0%
CC033	26	213.00	185.52	240.48	7	3~17	34.6%	34.6%	3.8%	0.0%
CC036	40	193.00	178.83	207.17	6	2~17	40.0%	37.5%	2.5%	0.0%
CC037	24	214.00	204.40	223.60	7	2~21	54.2%	54.2%	16.7%	0.0%
CC038	31	496.00	435.12	556.88	1	0~4	38.5%	38.5%	0.0%	0.0%
CC039	23	209.00	198.04	219.96	10	1~`8	47.8%	47.8%	0.0%	0.0%
CC041	26	244.00	237.65	250.35	7	1~15	46.2%	46.2%	0.0%	0.0%
CC042	21	177.00	168.03	185.97	13	7~22	57.1%	57.1%	0.0%	4.8%
CC051	23	396.00	325.60	466.40	3	1~6	53.8%	53.8%	0.0%	0.0%
CC057	18	215.00	206.68	223.32	6	2~14	61.1%	61.1%	0.0%	0.0%
CC059	17	287.00	264.14	309.86	7	3~10	50.0%	50.0%	0.0%	0.0%
CC080	20	352.00	299.41	404.59	2	1~7	50.0%	50.0%	0.0%	0.0%
FVB/N	20	184.00	175.23	192.77	8	2~13	35.0%	35.0%	0.0%	0.0%

Supplementary Table S2. List of SNPs and their location and corresponding gene, the p-values for each tumor phenotype at each SNP.

Supplementary Table S3. Candidate genes for each tumor phenotype.

Supplementary Table S4. Data for prognostic analysis in the METABRIC dataset.

Supplementary Table S5. Data for prognostic analysis in the TCGA-BRCA dataset.

Supplementary Table S6. Data for prognostic analysis in the GSE96058 dataset.

Supplementary Table S7. Data for drug response analysis in the I-SPY2 (GSE194040) dataset.

Supplementary Table S8. Human homologs of mouse candidate genes for each tumor phenotype.
